# Long Term Gene Expression in Human Induced Pluripotent Stem Cells and Cerebral Organoids to Model a Neurodegenerative Disease

**DOI:** 10.3389/fncel.2020.00014

**Published:** 2020-02-11

**Authors:** Ferid Nassor, Rafika Jarray, Denis S. F. Biard, Auriane Maïza, Dulce Papy-Garcia, Serena Pavoni, Jean-Philippe Deslys, Frank Yates

**Affiliations:** ^1^Service d’Etude des Prions et des Infections Atypiques (SEPIA), Institut François Jacob, Commissariat à l’Energie Atomique et aux Energies Alternatives (CEA), Université Paris Saclay, Fontenay-aux-Roses, France; ^2^CellTechs Laboratory, Sup’Biotech, Villejuif, France; ^3^Glycobiology, Cell Growth, Tissue Repair and Regeneration (Gly-CRRET), UPEC 4397, Université Paris Est Créteil, Créteil, France

**Keywords:** stem cells, cerebral organoid, neurodegenerative disease, fronto-temporal dementia, Alzheimer, IPS, disease modeling

## Abstract

Human brain organoids (mini-brains) consist of self-organized three-dimensional (3D) neural tissue which can be derived from reprogrammed adult cells and maintained for months in culture. These 3D structures manifest substantial potential for the modeling of neurodegenerative diseases and pave the way for personalized medicine. However, as these 3D brain models can express the whole human genetic complexity, it is critical to have access to isogenic mini-brains that only differ in specific and controlled genetic variables. Genetic engineering based on retroviral vectors is incompatible with the long-term modeling needed here and implies a risk of random integration while methods using CRISPR-Cas9 are still too complex to adapt to stem cells. We demonstrate in this study that our strategy which relies on an episomal plasmid vector derived from the Epstein-Barr virus (EBV) offers a simple and robust approach, avoiding the remaining caveats of mini-brain models. For this proof-of-concept, we used a normal tau protein with a fluorescent tag and a mutant genetic form (P301S) leading to Fronto-Temporal Dementia. Isogenic cell lines were obtained which were stable for more than 30 passages expressing either form. We show that the presence of the plasmid in the cells does not interfere with the mini-brain differentiation protocol and obtain the development of a pathologically relevant phenotype in cerebral organoids, with pathological hyperphosphorylation of the tau protein. Such a simple and versatile genetic strategy opens up the full potential of human organoids to contribute to disease modeling, personalized medicine and testing of therapeutics.

## Introduction

The prevalence of neurodegenerative diseases has amplified continuously over the years, notably due to an increasingly elderly population, and has become a major concern for many facets of society. The development of accurate modeling tools for human neurodegenerative diseases is therefore of vital interest for public health entities. With the development of human brain organoids, three-dimensional (3D) structures highly reminiscent of certain human brain regions, we are now a step closer to the accurate modeling of the human brain *in vitro* (Lancaster et al., 2013). Moreover, with the possibility of reprogramming patient cells to obtain induced pluripotent stem cells (iPSCs) while maintaining the original genetic characteristics of patients, we can now better decipher the events leading to the pathology. One of the initial hurdles identified regarding this model was its incompatibility with the modeling of events occurring late in the evolution of neurodegenerative diseases because of its embryonic/fetal nature (Camp et al., 2015). However, recent publications have shown that the cerebral organoid model is relevant for neurodegenerative diseases: specific markers, such as an imbalance of Aβ secretion, tau hyperphosphorylation and protein aggregation leading to the formation of amyloid fibrils have been described in organoids (Raja et al., 2016; Gonzalez et al., 2018; Pavoni et al., 2018). Nonetheless, one of the major issues for accurate modeling using patient-derived cerebral organoids is the control used for comparison. Indeed, because of the multiplicity of genetic and epigenetic factors when comparing cells from two individuals, there is a risk of missing crucial information (Vitale et al., 2012). To circumvent the issues surrounding this comparison, researchers have used additive (additional??) gene transfer strategies to express proteins of interest in a stable manner using retroviral approaches. Nonetheless, issues such as random integration, viral copy numbers, silencing, etc. hinder the potential of these applications in stem cells, especially in long term modeling approaches, where numerous cell passages and divisions are to be expected (Liew et al., 2007; Xia et al., 2007). Gene editing techniques based on CRISPR-Cas9 in human stem cells (embryonic or induced pluripotent), have been applied to create isogenic cell lines by adding or correcting a mutation (Grobarczyk et al., 2015; Li et al., 2015; Paquet et al., 2016). This novel approach circumvents the difficulty of finding isogenic controls, but the method requires both time and major resources, preventing a wide application of this technology. A different approach applicable to stem cells has been described by several authors with the use of episomal plasmid vectors derived from the Epstein-Barr virus (EBV; Ren et al., 2006; Thyagarajan et al., 2009). This plasmid allows the expression of a transgene able to replicate in the normal cell cycle due to the presence of OriP on the episome, without integration in the genome. The maintenance of the plasmid as an extrachromosomal element in a low-copy state has been attributed to the EBNA-1 region and its maintenance in the cell can be achieved through antibiotic selection (Lupton and Levine, 1985). In this report, the plasmid used was initially designed for gene silencing studies in tumor cell lines and has been extensively studied since (Biard et al., 2005). Existing approaches using EBV-based plasmids have been limited so far to the establishment of proofs of concept in stem cell lines, expressing fluorescent proteins, and do not show long term modeling approaches or validation for disease modeling.

This study describes the use of an EBV-based plasmid for pathological modeling using iPSCs and demonstrates the possibilities of its application in cerebral organoids to obtain isogenic cell lines that differ only in the expression of a single gene of interest. To do so, we developed a model based on a 4R tauopathy, the fronto-temporal dementia, which can be triggered with a mutation in the exon 10 of the MAPT gene, resulting in the conversion of a proline in a serine at the amino acid 301 in the tau protein (P301S; Hutton et al., 1998; Bugiani et al., 1999). This pathology constitutes a proof of concept, as tau protein can be tagged without affecting symptoms of its pathological progression, such as its aggregation capacity, as described by others (Frost et al., 2009). Notably, this pathology has been extensively described as a good modeling approach to understand the tauopathy underlying Alzheimer’s disease (Goedert et al., 2012). As described in patients, and elucidated in animal models, this 4R tauopathy manifests the following indications in the brain throughout the pathological progress: a synaptic loss, followed by hyperphosphorylation of the tau protein, leading to neurofibrillary degeneration, ultimately resulting in neuronal loss (Spillantini et al., 1998; Yoshiyama et al., 2007). Our goal in this work is to establish the capacity to model a neurodegenerative disease with episomal vectors on isogenic iPSCs differentiated in cerebral organoids in order to evaluate the development of a pathological state.

## Materials and Methods

### iPSCs Cell Culture

iPSCs were obtained by reprogramming BJ Fibroblasts obtained from ATCC (CRL-2522) in a previous study (Pavoni et al., 2018). Briefly, fibroblasts were reprogrammed using the Sendai virus reprogramming method as recommended by the manufacturer (Life Technologies). iPSCs were grown under feeder-free conditions on Matrigel-coated plates in E8 Flex Medium (Invitrogen). Seventy to eighty percent confluent iPSCs were passaged (ReleSR, Stem Cell Technologies) 1:5 and transferred to new wells in feeder-free conditions and incubated at 37°C, 5% CO_2_. Media were changed every 2 days and the cells split every 7 days. When cells possessed an EBV-based plasmid, the passaging medium used contained 0.5 μg/mL of puromycin in order to maintain the vector.

### EBV-Based Plasmid Generation

TauWT-YFP or TauP301S-YFP mutant open reading frames were introduced into puromycin-resistant EBV-based plasmid (pEBV) downstream of a CAG promoter to obtain the constructions of interest (Biard et al., 2005).

### iPSCs Electroporation

From a 35 mm dish, when iPSCs reached 70–80% confluence, cells were dissociated in a single-cell suspension using accutase. Electroporation was performed using an electro-square-porator (BTX 830, Harvard Apparatus) set on Low Voltage, using a 2 mm wide cuvette in PBS + 125 mM HEPES buffer with 1 pulse at 250 V of 1 ms, followed by three pulses at 50 V of 50 ms at 1 s interval. Ten microgram of plasmid DNA was used per electroporation. After electroporation, cells were plated on a Matrigel-coated plate with ROCK inhibitor (10 μM) present in the media for 24 h.

### Characterization of iPSCs

#### PCR Analysis of Gene Expression

Total RNA was isolated from iPSCs with the NucleoSpin RNA II kit (Macherey Nagel), in accordance with the manufacturer’s protocol. One microgram of each RNA sample was used to generate cDNA, with an iScript cDNA Synthesis Kit (Bio-Rad). RT-PCR was performed with the GoTaq DNA Polymerase kit (Promega). PCR products were separated by electrophoresis in a 1% agarose gel and analyzed with Gel Doc-it (UVP). RT-PCR for the 5′ coding region was performed with primers specific for OCT4 (sense primer 5′-AGCGAACCAGTATCGAGAAC-3′ and reverse primer 5′-TTACAGAACCACACTCGGAC-3′), SOX2 (sense primer 5′-AGCTACAGCATGATGCAGGA-3′ and reverse primer 5′-GGTCATGGAGTTGTACTGCA-3′), NANOG (sense primer 5′-TGAACCTCAGCTACAAACAG -3′ and reverse primer 5′-TGGTGGTAGGAAGAGTAAAG-3′), RPLP0 (sense primer 5′-CATTGCCCCATGTGAAGTC-3′ and reverse primer 5′-GCTCCCACTTTGTCTCCAGT-3′), REX1 (sense primer 5′-CAGTCCAGCAGGTGTTTGC-3′ and reverse primer 5′-GCATTCTATGTAACAGTCTGAGA-3′). The analysis was conducted at the population level at two different passages to confirm the presence and maintenance of the expression of pluripotency genes.

#### Teratoma Formation

iPSCs, whether expressing an EBV-based plasmid or not, were harvested at 70–80% confluence using ReleSR, collected into tubes, and centrifuged. The pellets were suspended in 50% Matrigel (Corning). Approximately 10^6^ cells were injected subcutaneously into NSG mice (NOD.Cg-Prkdc^scid^ Il2rg^tm1Wjl^/SzJ). Teratomas formed within 8–12 weeks. They were excised and fixed. Histological analysis was performed on sections stained with hematoxylin-eosin. One teratoma assay per cell line was performed. All animals were treated according to protocols approved by the local animal ethics advisory committee, registered with the French Ministry of Research and in accordance with French national regulations (national transposition of European directive 2010/63/CE). All animal experiments were approved by the Commissariat à l’énergie atomique et aux énergies alternatives (CEA), 92265 Fontenay-aux-Roses, France.

### Cerebral Organoid Formation

To generate cerebral organoids, we followed our previously described method (Pavoni et al., 2018) with minor modifications. Briefly, embryoid bodies (EBs) were formed by the hanging drop method with 10,000 cells per drop on the inside of a 10 cm dish lid. The lid was then placed on a dish filled with PBS to avoid evaporation. The dishes were transferred to an incubator at 37 C° with 95% relative humidity and 5% CO_2_. Media used in EB culture consisted of DMEM/F12 supplemented with KSR (20% v/v), MEM-NEAA (1×), 2-mercaptoethanol (0.1 mM), ROCK inhibitor (50 μM), dual SMAD inhibitors (SB431532 10 μM and LDN193189 100 nM) and bFGF (4 ng/mL). After 2 days, EBs were transferred to a 24-well plate and kept in this media up to day-6. On day 6, the culture medium was replaced with neural induction medium containing DMEM/F12-Glutamax medium supplemented with N2 supplement (1×), MEM-NEAA (1×) and antibiotic-antimycotic solution (1×). On days 11 and 12 of the protocol, cerebral organoids were embedded in growth factor-reduced Matrigel drops (BD Biosciences) and cultured in ultra-low attachment six-well plates (four cerebral organoids per well) in a differentiation medium containing DMEM/F12-Glutamax and Neurobasal medium (1:1 ratio) supplemented with N2 supplement (0.5×), B27 supplement without vitamin A (1×), 2-mercaptoethanol (0.2 μM), human insulin solution (2.5 μg/ml), MEM-NEAA (0.5×) and antibiotic-antimycotic solution (1×). On days 15 and 16, cerebral organoids were transferred to the same differentiation medium (the same as that described above with B27 supplemented with vitamin A) and the plates were placed in an orbital shaker (Infors) set at 70 rpm. The culture medium was replaced every 7 days.

### Western Blot

Cerebral Organoids were collected at 30 days. They were washed twice with PBS, snap-frozen in liquid nitrogen and stored at −80°C. To prepare the tissue homogenates, the samples were thawed partially on ice, resuspended (20% v/v) in ice-cold PBS containing protease (complete cocktail, Roche) and phosphatase inhibitors (50 mM NaF, 0.2 mM Na_3_3VO_4_) and lysed using a close-fitting rotating plunger. The protein concentration was estimated by the bicinchoninic acid method (BCA, Pierce). The supernatant was resuspended in SDS sample buffer (containing 33 mM dithiothreitol), boiled for 10 min, and electrophoresed through 4–12% Bis-Tris precast gels (Invitrogen). Proteins were transferred onto a PVDF (0.2 μm, Bio-Rad) membrane. Membranes were incubated with 5% non-fat milk for blocking. Phosphorylated tau (p-tau) was identified using the AT8 antibody and total tau (t-tau) using the TAU5 antibody. The blot was subsequently incubated with the appropriate horseradish peroxidase-conjugated secondary antibody. The blots were developed with ECL Plus reagents (Amersham GE Healthcare), and the immunoreactive bands were visualized by scanning with a Bio-Rad image analysis system. Each group of samples analyzed by western blot was collected from three independent cultures.

### Statistical Analysis

The data were plotted using GraphPad Prism software. Groups were compared *via* Student’s two-tailed *t*-test (two groups) or one-way analysis of variance (ANOVA; multiple groups).

## Results

### Generation of Transgene-Expressing iPSCs

**Figure 1:**
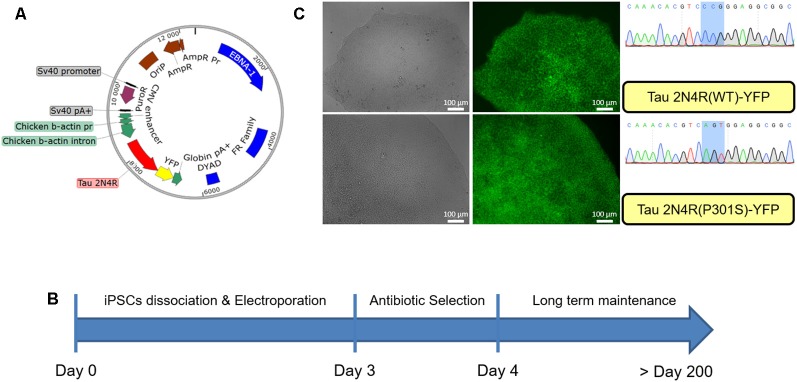
Generation of modified induced pluripotent stem cells (iPSCs) using an epstein-barr virus (EBV)-based plasmid. **(A)** Map of the EBV-based plasmid used in iPSCs, transgene expression under CAG promoter regulation, puromycin resistance under SV40 promoter regulation. **(B)** Workflow for EBV-based plasmid expressing iPSCs generation. **(C)** iPSCs colony after 30 passages post-electroporation expressing either wild type Tau 2N4R or mutated (P301S) form fused to YFP.

iPSCs obtained from reprogrammed fibroblasts in the laboratory were electroporated with EBV-based plasmids. As shown in Figure 1A the EBV-based plasmid expresses resistance to puromycin under an SV40 promoter allowing for an antibiotic selection of the cells. Forty-eight hours post-electroporation, puromycin was added for 24 h and cells then allowed to recover for up to 2 weeks, as shown in Figure 1B. After this point, the culture was maintained until colonies are ready for passaging with the expected morphological characteristics as shown in Supplementary Figure S1. At each passage, the media was supplemented with puromycin (0.5 μg/ml) for 24 h allowing for the maintenance of the episomal vector in the cells. We did not observe the repression of the reporter YFP gene up to 30 passages in the presence of puromycin (Figure 1C).

### Characterization of Transgene-Expressing iPSCs

**Figure 2:**
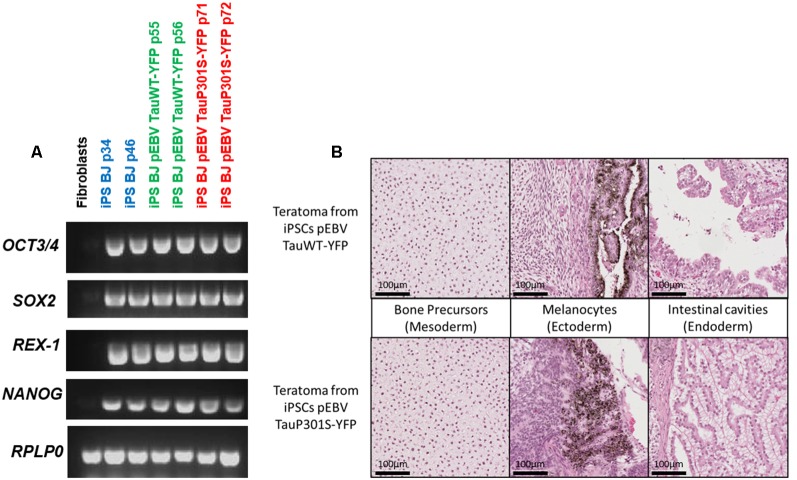
Pluripotency characterization of transgenic iPSCs.** (A)** Assessment of pluripotency by semi-quantitative PCR to confirm the expression of pluripotency markers in the control iPS line BJ and in EBV-based plasmid expressing lines. PCR analysis shows the expression of pluripotency markers OCT4, SOX2, NANOG and REX-1. BJ fibroblasts were used as a control and RPLP0 as a housekeeping gene. **(B)** H&E staining of a section of teratomas obtained from iPSCs expressing either Tau WT or P301S using an EBV-based plasmid showing the development of structures specific to the three germ layers.

The newly established iPSCs lines maintaining an EBV-based plasmid were characterized to verify whether the presence of the plasmid or the expression of the transgenes interferes with the phenotypical and functional characteristics of pluripotent stem cells. The pluripotency of iPSCs electroporated with the EBV-based plasmid expressing either TauWT-YFP or TauP301S-YFP fusion protein was investigated. This analysis was performed by assessing the expression of the endogenous pluripotency marker genes SOX2, OCT4, NANOG and REX-1 by RT-PCR. RPLP0 gene expression was used as an internal control for this assay. The pluripotency markers were strongly expressed in all iPSC clones but not in the initial fibroblasts as expected and shown in Figure 2A. We assayed the teratoma-forming potential of episome-transfected iPSC and carried out a histological analysis of tumors derived from these cells. These teratoma assays provided a clear evaluation of the impact on differentiation and proliferation of these iPSCs *in vivo* over a period of several weeks. Histological analysis of the tumors showed that these cells had differentiated into endodermal, ectodermal and mesodermal tissues (bone precursor, intestinal cavities and melanocytes) as shown in Figure 2B. The tissues were well-differentiated, without malignancy, in all the structures observed.

### Pathological Modeling Using Cerebral Organoids

Next, for pathological modeling, the cerebral organoid model was used as a method to obtain a heterogeneous composition of neural cells, representative of the complexity of the human brain. Cerebral organoids have already been used for their potential to model neurodegenerative phenotypes (Raja et al., 2016; Gonzalez et al., 2018). Furthermore, we have previously shown that chemical compounds added to the culture medium can induce an imbalance of the Aβ42/Aβ40 ratio comparable to that seen in human Alzheimer’s disease patients (Pavoni et al., 2018).

**Figure 3:**
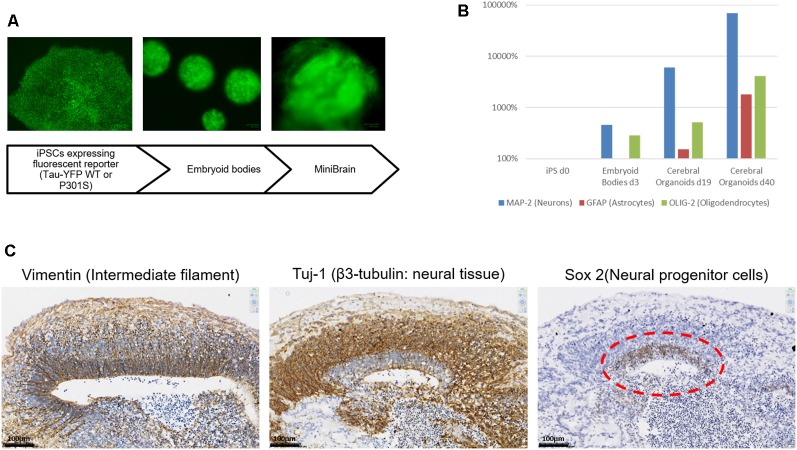
MiniBrain generation with modified iPSCs for pathological modeling. **(A)** Diagram showing the major steps of the MiniBrain protocol from iPSCs to cerebral organoids.** (B)** Real-time PCR analysis shows the expression of different neural markers at different time points in the cerebral organoid formation protocol, MAP-2 for neuron evaluation, GFAP for astrocyte evaluation and Olig-2 for oligodendrocyte evaluation. **(C)** Tissue sections of a 30-day cerebral organoid showing the development of neural rosettes with a radial organization, as shown with vimentin staining. Sox-2 staining reveals a neural stem cell niche, from which a neuronal network is developing as can be seen with the Tuj1 staining.

It was first ensured that the modifications introduced to the original Lancaster et al.’s (2013) protocol and the expression of transgenes on the EBV-based plasmid did not interfere with the differentiation steps of our model. *in vitro* differentiation was performed while avoiding antibiotic selection to avoid any toxicity. This choice was also guided by preliminary results showing that the number of divisions occurring to obtain an organoid was compatible with growth in absence of antibiotic selection, without losing the vector. As shown in Figure 3A, the differentiation pattern is not hampered by the presence of the episomal vector. Both expression markers and immunohistochemical characterization point towards an internal organization coherent with what has previously been described for the cerebral organoid by others (Lancaster et al., 2013). Real-time PCR analysis shows the presence of different neural cells (Neurons, Astrocytes and Oligodendrocytes) whose proportions increase during culture (Figure 3B). On the immunohistochemical sections, the development of a neural stem cell niche can be discerned developing outwards in neuronal cells supported in a radial organization as shown with laminin staining (Figure 3C).

**Figure 4:**
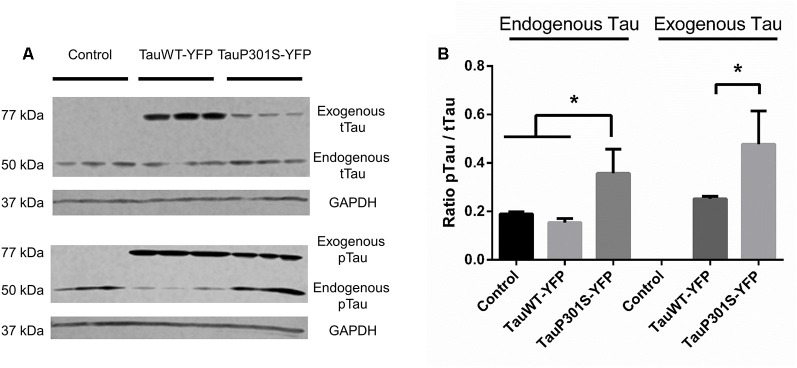
FTD pathological modeling using modified iPSCs expressing an EBV-based plasmid.** (A)** Western Blot analysis of total tau protein (tTau, TAU5 antibody) and phosphorylated tau protein (pTau, AT8 antibody). The expression of a fusion protein enables the endogenously expressed tau protein to be distinguished from the exogeneous form expressed using the EBV-based plasmid.** (B)** Ratio of phosphorylated tau protein over total tau against GAPDH for endogenous and exogenous tau. Statistical analysis: one-way analysis of variance (ANOVA; multiple groups). On charts *p < 0.05.

To verify the continuous presence of the episomally expressed forms of tau a western blot of organoids was performed after 30 days of culture. The DNA construct used, with a protein fused to a fluorescent reporter, enables the endogenous tau to be distinguished from the exogenous tau (i.e., the tau protein translated from the transgene carried by the episomal vector) by the differences in their sizes (addition of 27 kDa due to the YFP). As has been described by others (Yoshiyama et al., 2007), one of the first pathological symptoms of the frontotemporal disease is a hyperphosphorylation of the tau protein. Immunoblotting was therefore performed using the AT8 antibody, recognizing the first site to be phosphorylated of the tau protein in tauopathies, to assess the phosphorylation of the protein and one using TAU5 antibody, recognizing all tau isoforms, to have an estimation of total tau. As shown in Figure 4, there are no significant differences between control cells and the overexpression of Tau2N4RWT for the phosphorylation ratio of tau. However, from the P301S overexpression, hyperphosphorylation of the exogenous P301S mutant form compared to controls can be seen, as can hyperphosphorylation of the endogenous form of tau, which is not mutated. These results are also confirmed through IHC, which shows a significant change in the surface ratio of p-tau as shown in Supplementary Figure S2.

## Discussion

The novel approach described here shows the possibilities offered by an episomal vector for disease modeling using iPSCs in a long-term approach with matching isogenic controls. We have been able to express different proteins using this system and the versatility of the plasmid allows a promoter of interest to be chosen. Here a CAG promoter was used. However, because of the dissociation between the expression of antibiotic resistance and that of the protein of interest, the construct can easily be modified. The establishment of a modified cell line based on electroporation of an EBV-based plasmid has proven to be simple, fast and reliable.

As the episomal vector is extrachromosomal, it allows the expression of transgene without the usual risk of random insertion encountered with lentiviral approaches. In addition, the benefits of the episomal vector approach for other applications is demonstrable, notably in gene therapy (Ehrhardt et al., 2008).

This modeling approach in a cerebral organoid shows that the vector is maintained during differentiation, without measurable silencing and allows the development of a relevant phenotype associated with the disease. In the present case, the absence of measurable protein aggregation can be attributed to a longer development of the disease which provides a rare opportunity to observe the pathology at its source and at a cellular level. For further investigation, artificial aging of our model *in vitro* could be used, as many systems have been described by studying progeroid syndromes. For instance, an inducible progerin expression or a chemically induced telomerase manipulation might provide the necessary cues for modeling aging *in vitro* (Miller et al., 2013; Carrero et al., 2016; Vera et al., 2016).

The methodology used here allows for the development of a pipeline for the large-scale production of isogenic lines for studying genetic diseases with the differentiation in complex models, such as cerebral organoids. However, this same technique could be used in all models based on pluripotent stem cells, becoming the basis for full-body modeling. This, in turn, can allow the advantage to be taken off the full potential of human organoids to contribute to disease modeling, personalized medicine and testing of therapeutics.

## Data Availability Statement

The datasets generated for this study are available on request to the corresponding author.

## Ethics Statement

The animal study was reviewed and approved by Commissariat à l’Energie Atomique.

## Author Contributions

FN, RJ, DB, AM, and SP performed the experiments. FN, RJ, DP-G, J-PD, and FY analyzed the data. FN, RJ, J-PD, and FY conceived the study, wrote and revised the manuscript.

## Conflict of Interest

The authors declare that the research was conducted in the absence of any commercial or financial relationships that could be construed as a potential conflict of interest.
