# Supplementary material for: Long Term Gene Expression in Human Induced Pluripotent Stem Cells and Cerebral Organoids to Model a Neurodegenerative Disease
Source: Front Cell Neurosci. 2020 Feb 11;14:14. doi: 10.3389/fncel.2020.00014 (PMC7026130; doi:10.3389/fncel.2020.00014)
Supplement: Supplementary file 1 [file Data_Sheet_1.DOCX]

## S1 Fig. Workflow for iPSCs electroporation with an EBV-based plasmid


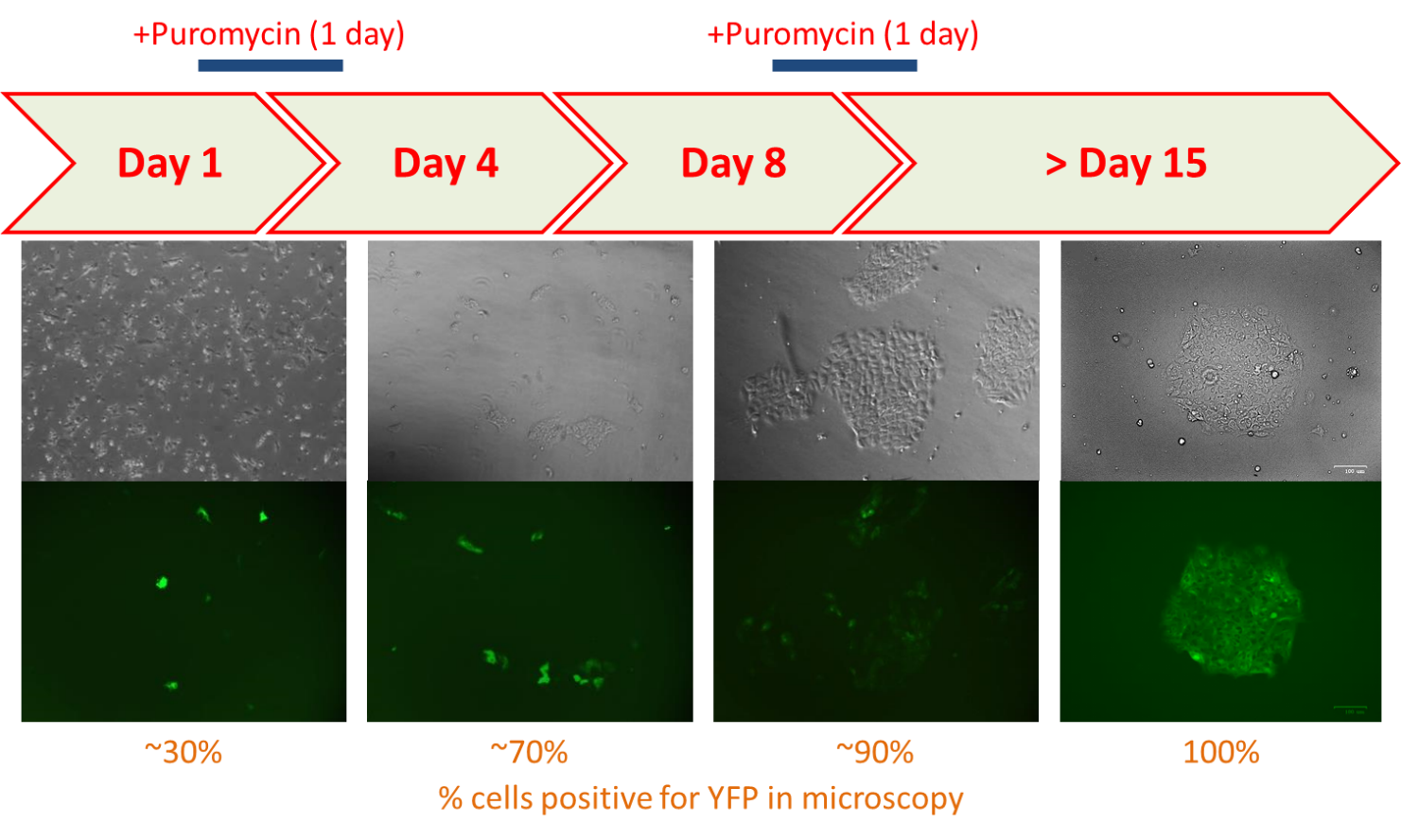


Microphotographs in Brightfield and Epifluorescent microscopy of iPSCs after electroporation and antibiotic selection. A visual estimation of the number of cells expressing the fused protein has been established during the recovery of the cells.

## S2 Fig. Evaluation of the hyperphosphorylation of the Tau protein due to the expression of the mutant form of Tau in 30-day cerebral organoids.


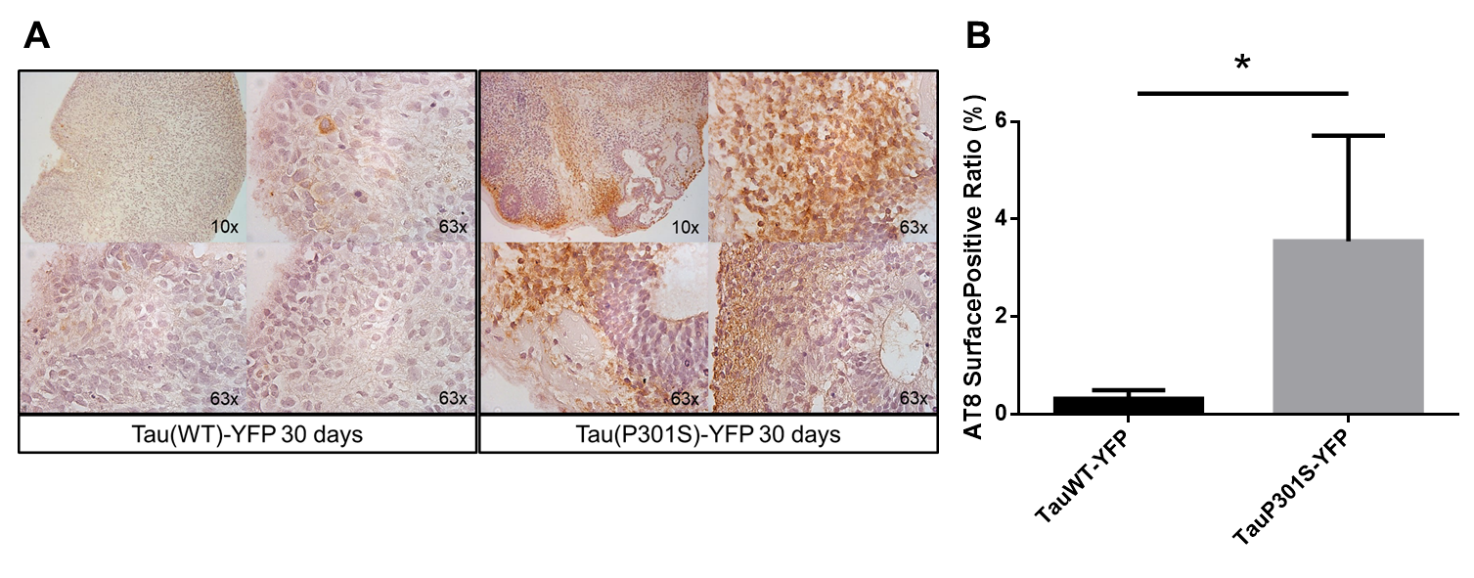


(A) Tissue sections stained of 30-day cerebral organoids stained with AT8 antibody expressing either WT form of Tau fused to YFP or mutated form (P301S) fused to YFP. (B) Analysis of the surface ratio of positive staining from 30-day cerebral organoids (n=3). Statistical analysis: Student’s two tailed t-test (two groups). On charts *: p < 0.05.
